# Antimicrobial uses for livestock production in developing countries

**DOI:** 10.14202/vetworld.2021.210-221

**Published:** 2021-01-25

**Authors:** Md. Zahangir Hosain, S. M. Lutful Kabir, Md. Mostofa Kamal

**Affiliations:** 1 Quality Control Laboratory, Department of Livestock Services, Savar, Dhaka-1343, Bangladesh; 2 Department of Microbiology and Hygiene, Bangladesh Agricultural University, Mymensingh-2202, Bangladesh

**Keywords:** antimicrobials, human health, livestock production, residue

## Abstract

Antimicrobial is an indispensable part of veterinary medicine used for the treatment and control of diseases as well as a growth promoter in livestock production. Frequent use of antimicrobials in veterinary practices may lead to the residue in animal originated products and creates some potential problems for human health. The presence of antimicrobial residues in animal originated foods may induce serious health problems such as allergic reaction, antimicrobial resistance (AMR), and lead to carcinogenic and mutagenic effects in the human body. The misuse or abuse of antibiotics in human medicine is thought to be a principal cause of AMR but some antimicrobial-resistant bacteria and their resistant genes originating from animals are also responsible for developing AMR. However, the residual effect of antimicrobials in feed and food products of animal origin is undeniable. In developing countries, the community is unaware of this residual effect due to lack of proper information about antibiotic usage, AMR surveillance, and residue monitoring system. It is imperative to reveal the current situation of antimicrobial use in livestock production and its impacts on public health. Moreover, the safety levels of animal feeds and food products of animal origin must be strictly monitored and public awareness should be developed against the indiscriminate use of antimicrobial in animal production. Therefore, the current review summarizes the literature on antimicrobial use in livestock production and its hazardous residual impacts on the human body in developing countries.

## Introduction

Antimicrobials are the substances produced naturally from living organisms or synthetically in the laboratory that can be administered orally, parenterally, or topically to kill or inhibit the growth of microorganisms [1]. Antimicrobials are generally used to treat, control, and prevent diseases. They are also used for non-therapeutic purposes such as feed proficiency enhancers and growth promoters in many countries worldwide [2-5]. Nowadays, almost 80% of all food-producing animals and birds receive medication for part or most of their lives [6]. Antimicrobials used in food animals account for 80% of the nation’s antimicrobial consumption annually in the USA [7]. In the past, the principal users of antimicrobials in food animals were developed countries such as the USA and some European countries [8]. At present, in developing countries of South and South East Asia region such as Bangladesh, Bhutan, India, Indonesia, Myanmar, Nepal, Sri Lanka, and Thailand, antimicrobials usage has rapidly increased in livestock [9-11]. In Bangladesh, about 94.16% of poultry farmers use antimicrobials [12] in their farms. The most commonly used antimicrobials for food animals are β-lactams, tetracyclines, aminoglycosides, lincosamides, quinolones, polypeptides, amphenicols, macrolides, and sulfonamides [13-15]. Globally, the average estimated annual consumption of antimicrobials in cattle, poultry, and the swine is 45 mg/kg, 148 mg/kg, and 172 mg/kg, respectively, and it is thought that global consumption of antimicrobials will increase 67% by the year 2030 [16].

Antimicrobials are predominantly used in treating animals and in ensuring good health. They are also used for preventing the spread of diseases in animals and poultry [17,18]. However, usage of antimicrobials in food-producing animals is debated. Repeated use of antimicrobials in food-producing animals for growth promotion, feed proficiency enhancement, and prophylaxis is believed to be a significant contributing factor for increasing antimicrobial resistance (AMR) [8,17,19]. Antimicrobials in food-producing animals may result in residues in food products such as milk, meat, eggs, and their byproducts which may eventually cause health-related problems. Allergy or hypersensitivity reaction, mutagenicity, and carcinogenicity could be severe forms of health issues. In less severe cases, alteration of microflora and the possible development of AMR may be developed [20-22].

Some commensal bacteria found in food-producing animals frequently propagates in fresh meat and milk products and may serve as reservoirs for resistant genes that could be transferred to pathogenic organisms in humans [20,23-25].

In developing countries, the antimicrobial residues typically occur due to indiscriminate and irrational use of antimicrobials in food animals without following the withdrawal period, extra-label dosages for animals, contamination of animal feed with the excreta of treated animals, and the use of unlicensed antibiotics [26]. An inappropriate antimicrobial drug used for humans is common in developing countries and is thought to be a significant reason for antimicrobial-resistant bacteria [27]. Poor-quality or sub-standard veterinary medicine is another important factor for AMR in livestock [28]. In developing countries, the demand for livestock products has increased significantly with the advancement of veterinary medical practices as well as the rapid growth of modern animal production systems. To meet this demand, overuse or improper usage of antimicrobials has happened. This increased usage of antimicrobials has threatened human health [12,28,29].

While antimicrobial residues in animal products can lead to various health risks including hypersensitivity reactions, cancer, bacterial resistance, toxicity, and teratogenicity, but the degree and relative impact of antimicrobials on human health still have not been well characterized in developing countries due to lack of proper information and surveillance system. Moreover, safe food has become an extremely important issue in international public health concern [30].

Therefore, based on the above facts the objectives of this review are:

- To reveal the current situation of antimicrobial use for animal production in developing countries.
- To describe the impact of improper use of antimicrobials on animal production.
- To develop strategies for alternatives to antimicrobial application in food animals, and
- To provide some recommendations to minimize the health-related problems of consumers resulting from antimicrobial residues in food-producing animals.

## Definitions of Antimicrobial Uses

### Therapy, control, prevention, and growth promoter

The National Committee for Clinical Laboratory Standards of USA has defined the terms to describe the uses of antimicrobials in the animal herd [31]. Therapy is the administration of an antimicrobial to a clinically diseased animal or group of animals. Control is the administration of an antibiotic to infected animals which exceed the baseline of morbidity and/or mortality. Prevention is the administration of an antibiotic to healthy animals which are thought to be at risk. Growth promotion is the administration of an antimicrobial, usually as a feed additive over a period of time to enhance the growth of animals by improving physiological performance.

### Maximum residue limit

Maximum residue level (MRL) of veterinary drugs is the maximum concentration or residue that results from the use of a veterinary antimicrobial or drug recommended by the Codex Alimentarius Commission to be legally permitted or recognized as acceptable in or on a food. The Codex Committee on residues of veterinary drugs in foods determines priorities for the consideration of residues of veterinary drugs in foods and recommends MRL for veterinary drugs. The MRL is based on the type and amount of residue considered to be without any toxicological hazard to human health as expressed by the acceptable daily intake. An MRL also considers public health risks as well as food technology issues. The unit used for MRL is expressed in milligrams per kilogram for solid products and milligrams per liter for liquids on a fresh weight basis [32].

### Withdrawal period

The withdrawal period is the time between the last doses of antimicrobials administered to the animal and when the level of residues in the tissues or products is lowered than or equal to the MRL. The withdrawal time may vary due to the chemical and physical properties of the antimicrobials and the route of administration [33].

### Pharmacodynamics of antimicrobial use

The main purpose of the use of antimicrobials is to treat infections and to eradicate the pathogen as quickly as possible with minimal adverse effects on the recipient. To achieve this goal, three basic conditions must be fulfilled by the antimicrobials [34].

- The antimicrobials should bind to a specific target-binding site on the microorganism.
- The concentration of the antimicrobials is to be sufficient to occupy the specific active sites on the microorganism and
- The antimicrobials should be occupied a sufficient number of active sites for an adequate period of time.

Simply, pharmacodynamics can be defined as the indexing of the total drug exposure in the serum or other body sites to measure the microbiological activity of an agent against the organisms [34]. The relationship between the concentration of antimicrobials and the retention time of that concentration at the active sites is termed as the area under the concentration-time curve (AUC), which is crucial to the life and death of the microorganism [35]. The extent of microbiological activity commonly known as minimum inhibitory concentration (MIC) is an important parameter in pharmacodynamics and the AUC/MIC ratio is thought to be a crucial point to explore the antimicrobial activity of an antimicrobial drug or agent [36]. Usually, the higher AUC/MIC ratio increases the probability of maximum eradication of the organism. Resistance can occur as a result of using low doses of antimicrobials to an organism that has higher MIC values [37]. Therefore, the use of higher AUC/MIC ratios is important to eradicate the organism as well as to minimize the risk of selection of resistant organisms. These basic principles of pharmacodynamics should be considered before using antimicrobials in animals [38].

## Scenario of Antimicrobial Uses

### Antimicrobial use in food animals

In recent years, the levels of animal product production and consumption have rapidly increased due to the demand of animal protein. Due to vast changes in food production and feeding regimens, the use of antimicrobials also increased in the livestock sector. It is estimated that livestock alone uses 50-80% of the total antibiotics produced in most of the developed countries [4]. China, Brazil, and the United States are the largest consumers of antimicrobials in livestock production [16]. The developing countries of South and South East Asia Region have developed intensive farming systems, leading to the rising of livestock population, and consumption of antimicrobials (Table-1) by food-producing animals [12,16,39-43]. The antimicrobial consumption is expected to rise significantly by 2030 in some developing countries such as Myanmar (205%), Indonesia (202%), Nigeria (163%), Peru (160%), and Vietnam (157%) due to increase in meat consumption [16]. In India, the national diet has changed due to increased consumption of meat by the young urban generation [44], which has resulted in intensive breeding of food animals with the routine use of antibiotics as growth promoters. In Bangladesh, about 94.16% of poultry farmers use antibiotics in their farms to control disease and enhancement of egg production [12] and more than 70% of the total quantity of antibiotics used in the food-producing animal manufactured by local pharmaceutical companies [45]. Most farmers in commercial poultry use antibiotics themselves directly from feed dealers or even directly from companies or suppliers. A major portion of antibiotics is sold to the customers or animal farm owners through over-the-counter. A study showed that more than 60% of farmers use antibiotics without the prescription of veterinarians [29].

**Table-1:** Total livestock population and the use of antimicrobials by food-producing animals in developing countries.

Country	Total livestock population including poultry (in million)	Use of antimicrobials	References
Bangladesh	425.91	94.16% poultry farms	[12]
Bhutan	1.17	Antimicrobial uses for animal production are limited in Bhutan.	[39]
India	1322.25	India accounts for 3% of global antibiotic consumption and it is estimated to increase by 82% by 2030.	[16]
Indonesia	2506.15	About −27% poultry farms and 81.4% of farmers routinely give antibiotics as a prophylactic	[40]
Myanmar	368.01	Most of the poultry producers	[41]
Nepal	99.19	About −62%poultry farms	[42]
Sri Lanka	22.39	Large amount of antibiotics are used in intensively managed animal husbandry in Sri Lanka	[43]
Thailand	308.40	9% pig farms	[40]

Source of data (livestock population): FAOSTAT, the FAO Statistics Division, 2020, www.fao.org/faostat/en

### Prophylactic and growth-promoting use of antimicrobials

Most prophylactic uses of antimicrobials involve mass medication of a group or flock of animals through feed or water. The antimicrobials registered for prophylactic use are categorized country wise and according to their value in human medicine. About 37% of antimicrobials used in animal production do not have similar drugs used for therapeutic purposes in humans [46]. Penicillin and tetracyclines are the most commonly prescribed antibiotics used for food animals [47], whereas for human therapeutic purpose antimicrobial drug categories as the macrolides, polymyxins, aminoglycosides, and third-generation cephalosporins (Table-2), which are also being used in food animals [8,19,48,49].

**Table-2:** World Health Organization antibiotic drug categories, their significance in human medicine, and some examples used in food animals.

Antimicrobial class	Significance in human therapeutics	Examples utilized in food animals
Aminoglycosides	Critically important	Gentamicin, neomycin
Macrolides and lincosamides	Critically important	Erythromycin, tilmicosin^a^, lincomycin^a^, tulathromycin^a^, tylosin^a^
β-Lactams	Critically important	Penicillin, ceftiofur^a^, amoxicillin
Fluoroquinolones	Critically important	Ciprofloxacin, danofloxacin^a^, enrofloxacin^a^
Tetracyclines	Highly important	Chlortetracycline, oxytetracycline, tetracycline
Streptogramins	Highly important	Virginiamycin^a^
Phenicols	Highly important	Florfenicol^a^
Sulfonamides	Highly important	Many sulfonamides
Polymyxins	Important	Colistin

a Exclusively veterinary application

Antibiotics used as growth promoters are often administered to animals in sub-therapeutic doses than for therapeutic applications which are likely to provoke more substantial pressure with regard to AMR emergence as it involves the repeated exposure of sub-lethal amounts of antibiotics to the bacteria. Moreover, some antimicrobials such as the ionophores and sulfonamides are used as coccidiostats for prophylactics purposes to prevent coccidiosis in poultry, and some coccidiostats are used in poultry food that possesses antibacterial characteristics [18]. In Bangladesh, nearly all dairy cows receive intra-mammary infusions of prophylactic doses of antibiotics following each lactation to prevent and control future mastitis [50]. However, about 90% of all antimicrobials used in farm animals are reported to be administered at the sub-therapeutic concentration [51] and a large portion of these are used as growth promoters to facilitate feed conversion ratio and disease control purposes [52].

## Risks Associated With the Use of Antimicrobials in Livestock Production

### Antimicrobial residues

Antimicrobials extensively used for food-producing animals in different counties of the world are – beta-lactams, tetracyclines, macrolides, sulfonamides, aminoglycosides, fluoroquinolones, lincosamides, and cephalosporins groups [13,52]. Recent reports have revealed that the use of large amounts of antimicrobial drugs could result in the deposition of antibiotics as residues in animal products [53,54]. Milk, meat, and other dairy products containing drug residues beyond the MRL may produce serious health problems to the consumers [26]. Although good quality milk, meat, and other related products are a prime need for maintaining proper public health [54], the presence of antimicrobial residues in these food items and their subsequent consumption may cause serious health problems to consumers including the development of AMR, hypersensitivity reaction, and cancer [22]. The consequences of such resistance are even more threatening where antibiotics become ineffective clinically for the treatment of illness. Food and Agriculture Organization (FAO)/World Health Organization (WHO) reported that antibiotic residues in edible animal products have grown beyond the permissible levels in developing countries [55]. The developing countries are at greater risk compared to developed countries due to poor detection facilities as well as lack of proper monitoring system of residues in foods considering the MRLs [29]. Indiscriminate and irrational use of antimicrobials in livestock production especially in small farming without following the withdrawal period is the main causes of residues in animal originated food products in developing countries [26]. Some studies demonstrated that antibiotics are deposited in the liver, kidney, muscle, and bones of poultry and livestock exceeding the MRL values [53]. A high percentage of poultry meat and eggs for human consumption were found to have antimicrobial residues such as amoxicillin, tetracycline, ciprofloxacin, and enrofloxacin [56]. A study performed at Chittagong region of Bangladesh on dairy and poultry farm which demonstrated that the tetracycline, amoxicillin, and ciprofloxacin residues were significantly higher in commercial farms compared to local and the concentration amoxicillin residue in milk and eggs were 56.16 μg/mL and 48.82 μg/g, respectively [26]. In another study, tetracycline, ciprofloxacin, enrofloxacin, and amoxicillin residues were found in poultry liver as 48%, 44%, 40%, and 42% cases, respectively, and the concentration of amoxicillin particularly in the liver was 16.92 μg/kg-152.62 μg/kg and in breast muscle was 45.38 μg/kg-60.55 μg/kg [57].

### AMR

Resistance to the antimicrobial drug is one of the most serious medical problems in the world [58], in which antimicrobial agent such as antibiotic is not effective for the treatment of infection due to the acquired resistance of bacteria to all available antibiotics. The improper antimicrobial drug used for humans is diffusive in developing countries and is a significant contributor to the growing public health threat of AMR-resistant bacteria [27]. In recent years, the indiscriminate use of antibiotics in animals is thought to be an important factor in developing AMR [29,58]. In Bangladesh, the most common reason for choosing an antimicrobial is personal experience and perception (68%), rather than the cultural sensitivity test which may due to lack of vet diagnostic facilities and the unwillingness of the veterinary personals [59]. Farmers’ wisdom about the use of antibiotics is another important determinant to provoke the problem of antibiotic resistance [60]. A study showed that most farmers have no clear idea about AMR and the withdrawal period of antibiotics [61]. Resistance to antibiotics includes – first, transfer of AMR pathogens through the food chain, and transfer of AMR genes from animal enteric flora to human pathogens. Second, there is a reduced efficacy of antibiotic therapy in animals colonized with resistant bacteria [59]. There is evidence that resistance in some human enteric pathogens has emerged due to the transfer of resistant bacteria or resistance genes from animals to humans through the food chain or through the contaminated environment [25,26,58]. The scientific reports that have published on multidrug-resistant bacteria isolated from food animals and products in developing countries are summarized in Table-3 [25,62-79].

**Table-3:** Prevalence of antibiotic-resistant bacteria isolated from food animals and products in developing countries.

Country	Type of specimens	Antimicrobial-resistant bacteria	Prevalence (%)	References
Bangladesh	Poultry	MDR *E. coli*	36.6	[62]
	Milk	MDR *C. jejuni*	57.1	[63]
		MDR *C. coli*	33.33	
	Poultry	MDR *C. jejuni*	49	[25]
		MDR *C. coli*	42	
	Milk	MDR *Salmonella* spp.	100	[64]
	Beef	MDR *Salmonella* spp.	66.67	
	Poultry meat	MDR *Salmonella* spp.	93.10	
	Milk	MDR *S. aureus*	12	[65]
	Poultry meat	MDR *S. aureus*	53.85	
	Egg	MDR *S. aureus*	90.91	
Bhutan	Pig fecal sample	MDR *E. coli*	2.4	[66]
India	Bovine	MDR *E. coli*	71.43	[67]
	Poultry	MDR *E. coli*	63.2	[68]
	Piglets	MDR *E. coli*	80	[69]
	Bovine	Shiga toxin producing *E. coli*	17	[70]
	Poultry	MDR *Salmonella* spp.	100	[71]
	Bovine	MDR *S. aureus*	20-30	[72,73]
Indonesia	Poultry	MDR *E. faecalis*	84.5	[74]
Myanmar	Poultry	MDR *Salmonella* spp.	52.2	[75]
Nepal	Buffalo and poultry meat	MDR *E. coli*	52.5	[76]
		MDR *Proteus* spp.	77.7	
		MDR *S. aureus*	40.0	
Sri-Lanka	Cattle, Pig and poultry	MDR *S. aureus*	65	[77]
Thailand	Poultry	ESBL-producing *S.* Typhimurium	77.3	[78]
	Pig	ESBL-producing *S.* Typhimurium	40.4	
	Pig	ESBL-producing *E. coli*	77	[79]
	Pork	ESBL-producing *E. coli*	61	
	Pork	MDR *A. baumannii* and *P. aeruginosa*	40	
	Poultry	ESBL-producing *E. coli*	40	
	Poultry meat	ESBL-producing *E. coli*	50	

MDR=Multidrug-resistant, ESBL=Extended spectrum beta-lactamase. *E. coli=Escherichia coli, C. jejuni=Campylobacter jejuni, C. coli=Campylobacter coli, S. aureus=Staphylococcus aureus, E. faecalis=Enterococcus faecalis, S.* Typhimurium=*Salmonella* Typhimurium, *A. baumannii=Acinetobacter baumannii, P. aeruginosa=Pseudomonas aeruginosa*

### Containment measures of AMR

The livestock sector is the largest consumer of antimicrobial and one of the important risk factors for developing AMR and other health-related problems in humans. However, the following strategies can be adopted to reduce the antimicrobial uses in livestock production and subsequently its impact on the human population.

### Development of an integrated and multi-sectorial action plan

The developing countries have minimal or no programs to monitor antimicrobial use or AMR in food animals, food products, and humans. Moreover, antimicrobials are extensively used in agriculture for growth-promotion, and the danger of AMR in the food chain is further neglected and under-estimated [80]. The majority of developing countries are not implementing adequate measures to prevent the spread of AMR from farm-to-fork thus, provoking a significant threat to global public health. To address the threat of AMR integrated health approach was endorsed by supranational entities, the coalition between the WHO, FAO, and World Organization for Animal Health (OIE) referred to as the “Tripartite Alliance.” The WHO, in collaboration with its tripartite partners, published the Global Action Plan on AMR to counteract this worldwide concern effectively [81]. Similarly, FAO launched its AMR strategy to support the implementation of the WHO’s Global Action plan in the food and agricultural sectors [82]. OIE also established principles and criteria for antimicrobial use and the list of antimicrobial agents of veterinary importance [83,84]. It is very important that every country should follow the guideline of OIE for using antibiotics in food-producing animals. The WHO’s Global Action Plan proposes solutions for effective containment of AMR from farm-to-fork that is applicable in both developed and developing nations. The five strategic objectives of the WHO’s Global Action Plan are – (i) heighten awareness and understanding on antimicrobial use and AMR, (ii) strengthen knowledge through surveillance and research, (iii) minimize infectious diseases, (iv) optimize rational antimicrobial use, and (v) mobilize resources, research, and development, to set out integrated prevention and containment measures of AMR in the food chain. It is, therefore, important to develop an integrated and multi-sectorial action plan following the Global Action Plan to reduce the uses of antimicrobials and AMR in food animals.

## One Health Approach

AMR is a complex, multifaceted problem that threatens human and animal health as well as the world economy resulting from the improper use of antibiotics. Globally, AMR is increasing [85] and it is thought that if the current trends continue, AMR will result in 10 million deaths per year from a wide range of infections by 2050 [86]. Various cases of AMR in humans have been reported to resistant microbes suspected of originating in livestock [87,88], which is mainly from asymptomatic infected livestock [88]. Transmission of resistant bacteria from livestock to humans can occur through the consumption of meat, direct contact with colonized animals, or manure spread in the environment [87]. Thus, the AMR is a multi-sectorial problem, and coordinated response of the human, animal, and environmental sectors should adopt a comprehensive approach such as that of one health is needed to combat the effect of AMR. Based on this concept, controlling the transmission of infection from animals and environment to humans, the government of different countries came up with the National One Health Strategy in 2012 [56]. Like other countries, in 2016, a One Health Secretariat was established at the Institute of Epidemiology, Disease Control and Research in Bangladesh with staff from three ministries (human health, animal health, and environment) and support from the development partners [89]. Although, improving awareness among professionals and practitioners across the sectors for consensus actions, and coordination among different sectors and ministries will be a big challenge, we need a more comprehensive approach [90] to move forward to response the health emergencies such as outbreaks of epidemics and pandemics including AMR. Therefore, one health approach may be an effective way to reduce the uses of antibiotics and subsequently the hazardous impact of antibiotics in livestock as well as humans.

## Development of Organic Production

Organic livestock production may be defined as a process that enhances the use of organic and biodegradable inputs from the ecosystem for animal nutrition, health, housing, and breeding [91]. Organic livestock production can be a simple model for the successful elimination of the non-therapeutic use of antimicrobials in livestock production. Certified organic production is also the best way to combat AMR and protect the health of farmworkers, communities, and consumers. Besides, organic farming may be an essential part of the future of the food systems, together with a dramatic change in the food culture and reduction of antimicrobials in food [92]. Some studies have demonstrated that organic farms harbor fewer antimicrobial-resistant organisms compared to conventional methods [93-95] and also reduce the usages of antibiotics [96]. In organic livestock production systems, the producers need to follow a set of standards. The standard will be strictly verified by the certification agencies authorized by respective governments [91,97]. A farm may be classified as organic if it meets the criteria provided in a set of guidelines known as “organic standards.” Organic farming can be practiced by any farmer who is willing to follow its principles and guidelines and if the food is to be marketed or traded, it must be certified by an accredited agency. Although, the organic agricultural movement has been started in some countries, its expansion has remained limited due to some core problems such as poor farmers, poor farmer knowledge of organic farming and its benefits, insufficiency of organic inputs, and poor marketing of organic foods [98]. However, some key points that need to be considered for organic farming by producers and stakeholders are given below:

- The origins of livestock – all livestock and their products that are sold or labeled as organic must be raised under continuous organic management from the last third of gestation or at hatching.
- Livestock feed – livestock feed must be 100% organic and cannot contain any plastic pellets, feces, or slaughter byproducts.
- Living conditions – an organic livestock producer must create and maintain living conditions that must accommodate the natural behavior of the livestock and provide access to outdoors, fresh air and sunlight, and access to suitable pastures for ruminants. Animals must be supplied with adequate nutrition and when kept indoors, they must have dry, clean bedding, and substantial ventilation.
- Waste management – manure produced by organic livestock must be handled to ensure that it will not contaminate crops, soil, or water with heavy metals, or pathogenic organisms, and managed in a way that maximizes nutrient cycling back into the environment.
- Healthcare – organic livestock producers require to establish preventive health-care practices which include selecting the appropriate type and species of livestock and providing adequate feed and appropriate environment that minimizes stress, disease, and parasites. The organic livestock producers cannot provide preventive antibiotics and they are encouraged to treat animals with appropriate protocols, including antibiotics and other conventional medicines when needed, but these treated animals cannot be sold or labeled as organic.
- Record keeping – organic livestock producers need to maintain records of the organic farm. These records are important to verify the organic status of the animals and the production, harvesting, and handling practices associated with them and their products. It is also important to demonstrate that the records must comply with the Organic Food Production Act in the equivalent legislation elsewhere.

If organic farming methods can follow in livestock production, this will drastically reduce the risk of infection and transmission within organic operations. Moreover, this organic livestock production will reduce the use of antimicrobials in livestock.

## Use of Alternative Growth Promoters

Alternatives to antibiotic growth promoters such as probiotics, prebiotic, symbiotic [99], organic acid (citric acid and acetic acid) [100], and bioactive compound (tannin and saponin) [101] are safe and have no negative impact on the environments that can be used for livestock production that improves the growth performance and immunity in livestock [99,102-104] that will dramatically reduce the uses of antimicrobials in livestock.

## Vaccine

Regular vaccination may be one of the most effective ways for disease prevention as it directly prevents bacterial infections and may reduce the use of antimicrobials in livestock production. Besides, indirect vaccination also provides herd immunity, which extends the protection to the unvaccinated livestock [105] and may lead to a significant decline in total antimicrobial drug use [106].

## Vitamins and Minerals Supplementation

Vitamins and minerals paly some important roles in maintaining the immune function and inflammatory responses in animal body [90]. Vitamin A and D, and some minerals such as Zinc (Zn), Iron (Fe), and Selenium (Se) have a significant impact on immune function [107]. A study revealed that dietary supplementation of Copper (180 mg/day)+Zn (300 mg/day) +Vitamin E (500 IU/day)+Se (6 mg/day)+Vitamin A (53,000 IU/day)+beta carotene (300 mg/day) during the past 2 months of gestation is beneficial for control of sub-clinical mastitis in dairy cattle [108]. Therefore, vitamins and minerals supplementation may reduce the overall use of antimicrobials in livestock production by improving the health status of animals.

## Selective Dry Cow Therapy (DCT)

The study revealed that cows receiving antimicrobial DCT [109] had a significantly higher cure rate (86.6%) than the cows that did not receive antimicrobial treatment (59.2%) at this time [110]. In DCT, the antibiotics prevent new infections as well as treat the existing infections. Therefore, DCT may lead to a decrease in the overall use of antimicrobials in dairy farms.

## Herbal Medicine

Use of herbal medicine in veterinary practices is old but it can be adapted in animal health care and this practice may reduce the uses of antibiotics and minimize the residual effect of antibiotic in the animal originated products [111].

## Strengthening the Regulatory Control of Antimicrobial Uses

The AMR is a very critical issue in the world, and the South East Asia Region countries have already developed some comprehensive laws and policies (Table-4) to tackle the problem [112,113,114] by reducing the indiscriminate use of antibiotic in livestock sectors and promoting the sustainable improvements in livestock production.

**Table-4:** Legislation and policies adopted by developing countries for monitoring and controlling the use of antimicrobials in animal production.

Country	Title of the legislation/policies
Bangladesh	National drug policy-2005
	Fish feed and animal feed act-2010
	National livestock development policy-2007
	National strategy for ARC-2011
	Road map of a national action plan for ARC
	The drug (control) ordinance-1982
Bhutan	Medicines rules and regulation-2012
	National action plan on antimicrobial resistance (draft)-2015
	National antimicrobial policy (draft)-2015
India	Advisory on use of antibiotics in food-producing animals-2014
	Drugs and Cosmetics Act-1945
	Bureau of Indian Standards, poultry feed specification, 5^th^ revision-2007
	Food safety and standards (contaminants, toxins, and residues) regulations-2011
	National livestock policy-2013
	National policy for containment of antimicrobial resistance-2011
Indonesia	Law No 18 on husbandry and animal health-2009
	The Animal Health and Animal Husbandry Law No. 18, 2009
	Regulation of the head of the agency of drug and food control-2013
Myanmar	Fisheries law directive 9.96 on general product standard-1996
Nepal	Drug act-1978
	Drug registration rules-1981
	National drug policy-1995
Sri Lanka	Animal Diseases Act No 59-1992
Thailand	Code of practice for control of the use of veterinary drugs-2009
	Drug ACT B.E.2510 (A.C. 1967) and its amendment-2001

ARC=Antimicrobial resistance containment

The major objective of these Acts and Rules is to monitor the quality of poultry and animal feed of both local and import origin and to check the adulteration and standard of feeds. Uses of some antimicrobials as a growth promoter and prophylactic have been banned according to these laws and policies, although some antibiotics are still present in livestock products. Strict regulation of the laws and policies should be followed to reduce the use of antimicrobials in animal production.

## Conclusion and Recommendations

The substantial use of antimicrobials in food-producing animals has a major role in generating antimicrobial residues and the development of a global burden of AMR, allergic reaction, mutagenicity, carcinogenicity, and even death in humans. In developing countries, failure to maintain the withdrawal period and indiscriminate use of antimicrobials in livestock result in the occurrence of residues in animal originated food products. Despite the extensive adoption of antimicrobial in food-producing animals, proper information about the patterns and quantity of use are not well organized. Hence, this review will be helpful to reveal the current and real scenario of antimicrobial uses for livestock production in developing countries. However, the comprehensive data summarize in this review suggest that food-producing animals are responsible for a significant proportion of antimicrobial uses in developing countries. Therefore, based on the above conclusion the following recommendations could be followed to reduce the antimicrobial uses in livestock and subsequently could be minimized the health-related threatens in humans.

- Before purchasing antimicrobials, labeled instructions, and consequences of its usage should be read carefully.
- Maintaining the proper withdrawal time for using the antimicrobials in food-producing animals.
- Indiscriminate uses of antimicrobial, especially at sub-therapeutic doses for prophylaxis in animals should be ceased.
- Strictly follow the scientific guidelines and precautions to minimize the antimicrobial residue in foods of animal origin.
- Data regarding the uses of antimicrobials in food-producing animals should be preserved properly with date and cause of treatment, name, and dosage of antimicrobial used, withdrawal time, etc.
- The use of antibiotics in animals by non-veterinarian should be discouraged.
- Proper biosecurity should be maintained to prevent infectious diseases in animals following good production and good management practices to reduce the antimicrobial use in animal production.
- Effective surveillance and monitoring of antimicrobial residues in milk and milk products, meat and meat products, egg, etc., should be continued by the regulatory authority.
- Overall, public awareness about the indiscriminate uses of antimicrobial in animals and its potential residual impacts on the human body should be developed.

## Availability of Data and Material

All data generated or analyzed during this review were from studies available in the public domain.

## Authors’ Contributions

MZH: Conceptualized and designed the review. MZH and MMK: Collected the literatures. MZH: Analyzed the data. MZH and MMK: Drafted the manuscript. SMLK and MMK: Edited and finalized the manuscript. MMK: Collected funds. All the authors read and approved the manuscript.
